# Factors associated with psycho-behavioral problems among 100 children with phenylketonuria aged 6–18 years

**DOI:** 10.1186/s13023-025-03824-y

**Published:** 2025-06-11

**Authors:** Mei Xue, Ming Shen, Shunan Wang, Bo Pang, Xiaoqian Zhang, Kening Chen, Zhixin Zhang, Wenquan Niu

**Affiliations:** 1 Graduate School, Beijing University of Chinese Medicine, Beijing, China; 2 Department of Pediatrics, China-Japan Friendship Hospital, Beijing, China; 3 Chinese Academy of Medical Sciences & Peking Union Medical College, Beijing, 100005 China; 4 Institute of Clinical Medical Sciences, China-Japan Friendship Hospital, No.2 Yinghua East St., Chaoyang District, Beijing, 100029 China; 5 Center for Evidence-Based Medicine, Capital Institute of Pediatrics and Capital Center for Children’s Health, Capital Medical University, Beijing, China

**Keywords:** Phenylketonuria, Rare disease, Children, Factors, Psycho-behavioral problems

## Abstract

**Background:**

Phenylketonuria (PKU) is a rare disease. Children who are diagnosed with PKU often encounter psycho-behavioral difficulties, which can significantly impact quality of life and social integration. The aim of this study was to evaluate the prevalence of psycho-behavioral difficulties and explore potential factors associated with their occurrence in PKU children aged 6–18 years.

**Methods:**

From May 2022 to May 2024, 100 children with PKU were recruited using a questionnaire-based survey. Data were analyzed using the STATA (version 18.0) and R programming language (version 4.3.3).

**Results:**

25% of children aged 6–18 years with PKU exhibited psycho-behavioral problems. Significant factors associated with psycho-behavioral problems in study children included body mass index (multi-adjusted odds ratio, 95% confidence interval, P: 1.135, 1.010–1.276, 0.033), age (3.169, 1.024–9.804, 0.045), pregnancy order (0.143, 0.033–0.607, 0.008), delivery order (0.041, 0.004–0.373, 0.005), mode of disease diagnosis (5.730, 1.935–16.963, 0.002), and dietary therapy pressure (3.321, 1.083–10.181, 0.036). Based on these significant factors, a nomogram model was constructed with descent prediction capability and accuracy.

**Conclusions:**

We identified six factors closely associated with psycho-behavioral problems in PKU children, offering insights into risk profiles underlying these problems and guiding the formulation of effective prevention strategies.

## Introduction

Phenylketonuria (PKU), an inherited metabolic disorder affecting approximately 1 in 10,000 to 1 in 15,000 individuals globally [1], is characterized by the deficiency in phenylalanine hydroxylase (PAH) enzyme, which is pivotal for the metabolism of amino acid phenylalanine. The subsequent accumulation of phenylalanine in the bloodstream and brain can lead to a spectrum of neurodevelopmental problems if allowed to proceed uncurbed, including severe intellectual disability and psycho-behavioral complications [2, 3]. Despite the appearance of neonatal screening and dietary management, children with PKU may still face psycho-behavioral issues that can impact quality of life and social integration [4, 5].

Growing evidence underscores the significance and necessity of focusing on psycho-behavioral problems in PKU. Currently, many existing studies on this subject are limited by small sample sizes and mainly targeting the metabolic aspects of PKU, such as dietary management and biochemical monitoring [6, 7], and the relevant psychological and social implications are rarely considered in the literature. The authors of existing studies have called for more extensive research on psycho-behavioral difficulties among children with PKU, which can help gain a more comprehensive understanding of this condition and guide the formulation of effective prevention strategies.

Prior studies have raised a variety of psycho-behavioral challenges faced by children with PKU, including attention deficit, mood disorders, and social cognitive deficits [2, 8–12]. In fact, PKU exhibits high inter-individual variability, which remarkably challenges the specificity of neurocognitive and behavioral assessments [13]. Hence, addressing the psycho-behavioral problems in PKU is not only a matter of urgent clinical need, but a vital step forward the broader field of genetic and metabolic disorders. Emerging evidence suggests a bidirectional relationship where phenylalanine fluctuations disrupt neurotransmitter synthesis, yet triggering chronic stress responses that exacerbate executive dysfunction [14–16], which creates a vicious cycle of metabolic dysregulation and psychological impairment. However, there is no definite consensus on how many factors and which factors are actually responsible for the development of psycho-behavioral problems in PKU.

This study aimed to explore potential factors relating to birth history, disease-related elements, metabolic control, and family-related aspects that were associated with the significant risk of psycho-behavioral problems in PKU children aged 6–18 years. Psycho-behavioral problems in this study refer to a range of psychological and behavioral problems that include, but are not limited to, attention deficits, mood disorders (such as anxiety and depression), social cognitive deficits, and conduct problems according to the Conners Parent Symptom Questionnaire (PSQ) and the Hospital Anxiety and Depression Scale (HADS).

## Methods

### Study design

This is a cross-sectional survey conducted from May 2022 to May 2024 in accordance with ICMJE [17] guidelines, and the protocols of this study were approved by the Ethics Committee of China-Japan Friendship Hospital (Approval no. 2024-KY-081).

### Study participants

The study participants consisted of PKU children from the pediatric clinic of China-Japan Friendship Hospital, and their parents or guardians provided signature consent to the participation of this survey, with opt-out clauses in our consent form. Study participants were restricted to children aged 6–18 years, who had received a definitive diagnosis of PKU from a qualified clinician. Participants were excluded if they met any of the following criteria: (i) BH4 deficiency and DNAJC12 biallelic mutation; (ii) unavailable reliable medical history; (iii) prior diagnoses of familial or hereditary mental health disorders; (iv) complicated with other chronic diseases that may precipitate psycho-behavioral problems.

### Data collection

Data were collected by self-designed questionnaires. To ensure the reliability of our questionnaires, questionnaires were distributed in small samples before formal circulation, and had a reliability coefficient (alpha) over 0.85.

Contents in our questionnaires included the following parts: (i) demographic information: age, sex, height, weight, body mass index (BMI), and region; (ii) fetal and neonatal factors: birth length, birth weight, delivery mode, pregnancy order, and delivery order; (iii) family-related factors: maternal education level, paternal education level, and family income; (iv) disease-related information: disease diagnosis mode, age of initial treatment (month), highest phenylalanine before treatment, tyrosine, recent phenylalanine, average phenylalanine per year, diet therapy pressure, follow-up period, and control situation; specifically, phenylalanine and tyrosine levels were determined using high-performance liquid chromatography (HPLC) analysis performed at the clinical laboratory of our institution; (v) psycho-behavioral part: the Conners Parent Symptom Questionnaire (PSQ) and the Hospital Anxiety and Depression Scale (HADS) used to evaluate the psycho-behavioral status.

Dietary therapy pressure was defined as the presence of ≥ 2 of the following domains persisting > 6 months, based on clinical observations of patients exhibiting behavioral/emotional manifestations related to dietary management: (i) Precision pressure: stress from meticulously calculating phenylalanine intake to avoid metabolic dysregulation; (ii) Social restrictions: limitations on social activities due to reliance on special medical foods; (iii) Accessibility anxiety: psychological distress related to procuring medical foods (e.g., cost, availability); (iv) Nutritional imbalance fears: worries about long-term micronutrient deficiencies despite adherence. Above items were developed through iterative clinical consultations with metabolic dietitians and pediatric physicians over 5 years of PKU patient care. 

### Definitions of PKU and psycho-behavioral problems

In this study, PKU was defined according to official clinical practice guidelines [18]. Psycho-behavioral problems were assessed using the Parent Symptom Questionnaire (PSQ) [19] and the Hospital Anxiety and Depression Scale (HADS) [20]. Specifically, the PSQ evaluates six domains (conduct problems, learning problems, psychosomatic issues, impulsive-hyperactivity, anxiety, and a hyperactivity index) through 48 parent-reported items scored 0–3. Domain-specific scores were calculated by averaging item responses (range: 0–3), with scores > 1.5 (two standard deviations above population norms) indicating abnormality. The HADS, comprising anxiety (HADS-A) and depression (HADS-D) subscales (7 items each), categorizes symptom severity as 0–7 (none), 8–10 (possible), and 11–21 (clinically significant). Children were classified into four severity tiers: without psycho-behavioral (0 abnormal PSQ domain and HADS subscale score 0–7), slight (1 abnormal PSQ domain or HADS subscale score 8–10), moderate (2 abnormal PSQ domains or 1 abnormal PSQ domain with HADS subscale score 8–10), and severe (≥ 3 abnormal PSQ domains or concurrent HADS score > 8–10 with ≥ 2 abnormal PSQ domains). This stratification integrated both the breadth of behavioral dysfunction (PSQ domain counts) and the intensity of emotional symptoms (HADS severity thresholds) to reflect clinical multidimensionality.

### Definitions of other items

Delivery modes included vaginal delivery and caesarean section. Gestational age was used to distinguish term from preterm. Birth body length (to the nearest 0.1 cm) was reported by the parents or guardians of participant students. Pregnancy order and delivery order meant the times of pregnancy and bearing birth, respectively. Family education was the highest level of education of parents and was categorized as master’s degree or above, bachelor’s degree, and high school degree or below. Family income was categorized as ≥ 100,000 RMB (~ 14,000 USD, based on the 2023 average exchange rate), 50,000–100,000 RMB (~ 7,000–14,000 USD), and < 50,000 RMB (< 7,000 USD).

Disease diagnosis mode included neonatal screening and clinical diagnosis. Children diagnosed through neonatal screening received early treatment, while those diagnosed clinically were identified at an older age due to the emergence of symptoms (e.g., vomiting, eczema, and yellowing of hair). The age of initial treatment was measured in months. Average Phe per year was categorized as 2–6 mg/dL, 6–10 mg/dL, and > 10 mg/dL. Follow-up period included 4 times a year and less than 4 times a year. To assess long-term metabolic control, the average annual phenylalanine concentration was calculated for each participant. Based on these average annual phenylalanine levels, metabolic control was categorized into two groups. Well-controlled metabolic status was defined as phenylalanine levels ranging from 2 to 6 mg/dL for participants aged < 12 years and from 2 to 10 mg/dL for those aged ≥ 12 years; poorly controlled metabolic status was defined as phenylalanine levels exceeding 6 mg/dL for participants aged < 12 years and exceeding 10 mg/dL for those aged ≥ 12 years.

### Sample size estimation

The sample size was estimated based on the events per variable (EPV) metric [21, 22], a widely accepted method in statistical analyses. Given our intention to include 5–7 predictor variables (such as metabolic control indices, baseline features, and birth-related information) and set the EPV to 10, baseline requirement of 50–70 subjects was derived. To mitigate potential 20% attrition rates common in pediatric metabolic disorder cohorts, enrollment was expanded to 100 participants.

### Quality control

The selection of psycho-behavioral assessment tools, the Conners Parent Symptom Questionnaire (PSQ) and the Hospital Anxiety and Depression Scale (HADS), was conducted in collaboration with a clinical psychologist. All researchers completed standardized training for tool administration under the supervision of this psychologist to ensure protocol compliance. The study design and interpretation of results were reviewed by an interdisciplinary panel including a child psychologist. Ethical approval was granted by the institutional ethics committee, which included psychology experts.

To ensure data accuracy and reliability, two experienced outpatient physicians guided both parents and children through questionnaire completion, addressing ambiguities, and ensuring completeness under close supervision. Discrepancies in responses were resolved by a senior physician, with input from a second guardian if needed. Double data entry was independently conducted by two researchers (M.X. and B.P.) and adjudicated by a third (M.S.) to rectify inconsistencies. Physicians also conducted follow-up contacts to clarify missing or aberrant data and requested resubmissions when necessary, thereby maintaining rigorous quality control throughout the data collection process.

### Statistical analyses

Data were analyzed by using the STATA software (version 18.0) and R programming environment (version 4.3.3). The expression of continuous variables was mean (standard deviation) if no deviation from normal distributions tested by the skewness-kurtosis test, and median (interquartile range) otherwise. The expression of categorical variables was count (percent). Between-group comparisons (PKU children with versus without psycho-behavioral problems) of variables were conducted using the t-test for parametric data, the χ^2^ test for categorical data, and the rank-sum test for non-parametric data, where appropriate.

Correlation between predictive variables and specific problem scores were analyzed using Spearman’s rank-order correlation coefficients (r), with statistical significance determined through two-tailed nonparametric testing at an α of 5%, following evaluation of variable distribution assumptions and ordinal/continuous data compatibility.

Potential factors associated with different levels of psycho-behavioral problems in children with PKU were investigated using univariate and multivariate Logistic regression analyses. In multivariate analysis, sex, region, family income, maternal education level, and paternal education level in multivariable models were controlled. Effect-size estimates are expressed as odds ratio (OR) and 95% confidence interval (95% CI).

The enhancement in predictive accuracy achieved through the incorporation of significant factors into the basic model was comprehensively evaluated from both calibration and discrimination perspectives. From calibration aspect, the Akaike Information Criterion (AIC) and Bayesian Information Criterion (BIC) were leveraged to assess the degree of congruence between the predicted probabilities, incorporating these factors, and the actual observed risks. Furthermore, these criteria served to evaluate the overall suitability and goodness-of-fit of the refined risk model. From discrimination aspect, the Receiver Operating Characteristic (ROC) curve analysis for both the original and modified models was conducted.

Finally, a predictive nomogram for risk assessment was developed, incorporating key influential factors to improve its utility in clinical settings and public health initiatives. This model was created using the R programming environment (version 4.3.3).

## Results

### Baseline characteristics

**Fig. 1:**
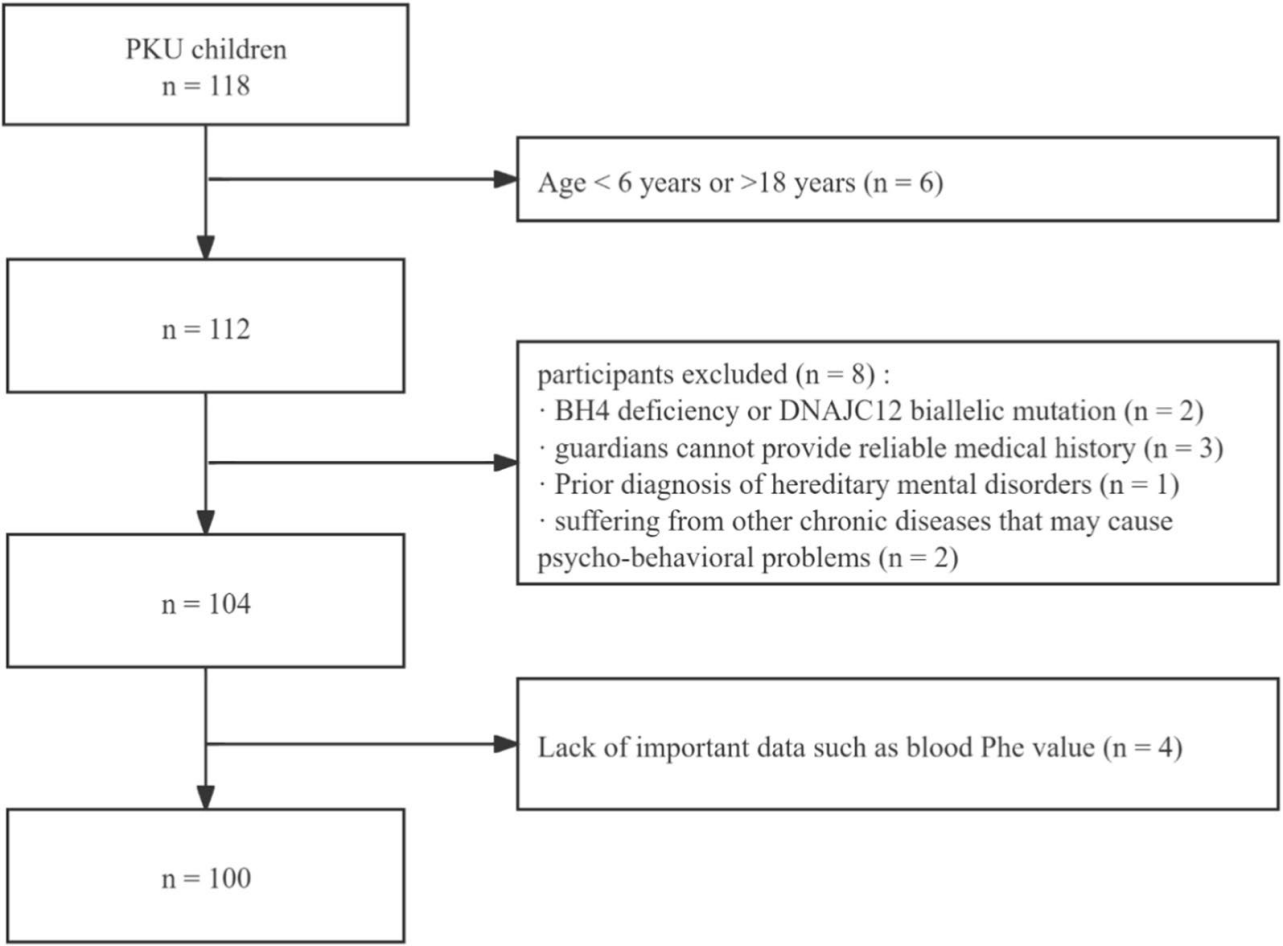
Flow chart illustrating the selection process of study participants

Detailed selection procedure is shown in Fig. 1. After rigorous screening to eliminate unqualified questionnaires, data from 100 children diagnosed with PKU, comprising 54 girls and 46 boys, were analyzed, with 25 children exhibiting psycho-behavioral problems, reflecting a prevalence rate of 25% among PKU children. Our study included 73 patients diagnosed through newborn screening and 27 patients diagnosed clinically. Among PKU children with psycho-behavioral problems, the age of diagnosis was 1 (1, 1) month for newborn-screened children and 28.5 (17.5, 42) months for clinically diagnosed children. Detailed baseline characteristics of all participating children are presented in Table 1.

**Table 1:** The baseline characteristics of study children

Characteristics	Children without Psycho-behavioral problem	Children with psycho-behavioral problem	*p*
	(n = 75)	(n = 25)	
**Demographic information**			
Age (years)			0.003
6–12	59 (78.7)	12 (48.0)	
12–18	16 (21.3)	13 (52.0)	
Sex			0.247
Boys	32 (42.7)	14 (56.0)	
Girls	43 (57.3)	11 (44.0)	
Height (cm)	136 (128, 155)	138.5 (126, 155)	0.939
Weight (kg)	32 (25, 46)	39 (28, 55)	0.200
BMI (kg/m^2^)	16.8 (15.2, 20.7)	19.5 (16.0, 22.2)	0.118
Region			0.006
City	58 (77.3)	12 (48.0)	
Country	17 (22.7)	13 (52.0)	
**Fetal and neonatal factors**			
Birth length (cm)	50 (50, 51)	50 (49, 50)	0.036
Birth weight (kg)	3200 (2900, 3500)	3000 (2650, 3400)	0.159
Delivery mode			0.096
Vaginal delivery	43 (57.3)	19 (76.0)	
Cesarean section	32 (42.7)	6 (24.0)	
Pregnancy order	1 (1, 2)	1 (1, 1)	0.034
Delivery order	1 (1, 2)	1 (1, 1)	0.005
**Family-related factors**			
Maternal education level			0.258
High school degree or below	37 (49.3)	16 (64.0)	
Bachelor’s degree	33 (44.0)	9 (36.0)	
Master’s degree or above	5 (6.7)	0 (0)	
Paternal education level			0.376
High school degree or below	39 (52.0)	17 (68.0)	
Bachelor’s degree	32 (42.7)	7 (28.0)	
Master’s degree or above	4 (5.3)	1 (4.0)	
Family income (USD per year)			0.335
< 7,000	23 (30.6)	8 (32.0)	
7,000–14,000	26 (34.7)	12 (48.0)	
> 14,000	26 (34.7)	5 (20.0)	
**Disease-related information**			
Disease diagnosis mode			< 0.001
Neonatal screening	62 (82.7)	11 (44.0)	
Clinical diagnosis	13 (17.3)	14 (56.0)	
Age at diagnosis (months)			< 0.001
Neonatal screening	1 (1, 2)	1 (1, 1)	
Clinical diagnosis	42 (30, 133)	28.5 (17.5, 42)	
Age of initial treatment (months)	2 (1, 19)	1 (1, 17)	0.599
Highest Phe before treatment	20 (11, 31)	22 (19, 24)	0.454
Recent Phe	7.9 (5.85, 12)	6 (3.63, 12.75)	0.245
Average Phe per year (mg/dL)			0.339
2–6	32 (42.7)	11 (44.0)	
6–10	28 (37.3)	6 (24.0)	
> 10	15 (20.0)	8 (32.0)	
Diet therapy pressure			0.007
No	64 (89.3)	15 (60.0)	
Yes	11 (14.7)	10 (40.0)	
Follow-up period			0.899
≥ 4 times a year	53 (70.7)	18 (72.0)	
< 4 times a year	22 (29.3)	7 (28.0)	
Control situation			0.817
Well control	38 (50.7)	12 (48.0)	
Poor control	37 (49.3)	13 (52.0)	
PSQ scores			
Conduct problems	0.17 (0.16, 0.41)	0.83 (0.50, 1.08)	< 0.001
Learning problems	0.75 (0.25, 1.00)	1.75 (1.25, 2.00)	< 0.001
Psychosomatic issues	0.20 (0, 0.20)	0.20 (0, 0.60)	0.386
Impulsive-hyperactivity	0.50 (0.25, 0.75)	1.25 (1.25, 1.75)	< 0.001
Anxiety	0.25 (0, 1.00)	1.00 (0.50, 1.25)	< 0.001
Hyperactivity index	0.50 (0.30, 0.70)	1.30 (0.90, 1.50)	< 0.001
PSQ detection rate			
Conduct problems	0 (0.0)	2 (8.0)	0.013
Learning problems	3 (4.0)	17 (68.0)	< 0.001
Psychosomatic issues	0 (0.0)	0 (0.0)	> 0.05
Impulsive-hyperactivity	0 (0.0)	8 (32.0)	< 0.001
Anxiety	1 (1.33)	4 (16.0)	0.004
Hyperactivity index	0 (0.0)	6 (24.0)	< 0.001
HADS scores			
Anxiety	3 (2, 5)	9 (5, 10)	< 0.001
Depression	3 (1, 5)	8 (6, 11)	< 0.001
HADS detection rate			
Anxiety			< 0.001
No symptoms	73 (97.3)	9 (36.0)	
Possible symptoms	2 (2.7)	12 (48.0)	
Presence of symptoms	0 (0.0)	4 (16.0)	
Depression			< 0.001
No symptoms	71 (94.7)	9 (36.0)	
Possible symptoms	4 (5.3)	8 (32.0)	
Presence of symptoms	0 (0.0)	8 (32.0)	

Data are expressed as median (interquartile range) or count (percent). P value was calculated by the rank-sum test or the χ^2^ test, where appropriate

*BMI* body mass index

### Identification of contributing predictors

Baseline variables shown in Table 1 were selected based on clinical relevance or with a *P* value < 0.05 on univariate analysis. After adjusting for sex, region, income, paternal education level, and maternal education level, six factors (BMI, age, pregnancy order, delivery order, disease diagnosis mode, and diet therapy pressure) were significantly associated with psycho-behavioral problems in children with PKU, as shown in Table 2. For example, the significant odds of having psycho-behavioral problems was 1.135 (95% CI: 1.010–1.276, *P* = 0.033) for BMI, 3.169 (95% CI: 1.024–9.804, *P* = 0.045) for age, 0.143 (95% CI: 0.033–0.607, *P* = 0.008) for pregnancy order, 0.041 (95% CI: 0.004–0.373, *P* = 0.005) for delivery order, 5.730 (95% CI: 1.935–16.963, *P* = 0.002) for disease diagnosis mode, and 3.321 (95% CI: 1.083–10.181, *P* = 0.036) for diet therapy pressure.

**Table 2:** Identification of potential factors responsible for psycho-behavioral problems in PKU children

Significant variables	Unadjusted model					Multivariable adjusted model*				
	OR	95% CI			*p*	OR	95% CI			*p*
BMI	0.950	0.927	to	0.972	**< 0.001**	1.135	1.010	to	1.276	0.033
Age	3.994	1.530	to	10.428	**0.005**	3.169	1.024	to	9.804	**0.045**
Pregnancy order	0.302	0.092	to	0.992	**0.049**	0.143	0.033	to	0.607	**0.008**
Delivery order	0.098	0.013	to	0.749	**0.025**	0.041	0.004	to	0.373	**0.005**
Highest Phe before treatment	0.961	0.942	to	0.979	** < 0.001**	0.993	0.956	to	1.031	0.726
Recent Phe	0.902	0.859	to	0.947	**< 0.001**	0.985	0.897	to	1.081	0.750
Age of initial treatment	0.971	0.948	to	0.995	**0.018**	0.986	0.964	to	1.007	0.199
Delivery mode	0.442	0.257	to	0.758	**0.003**	2.307	0.785	to	6.783	0.129
Disease diagnosis mode	6.069	2.254	to	16.343	**< 0.001**	5.730	1.935	to	16.963	**0.002**
Diet therapy pressure	0.504	0.355	to	0.716	**< 0.001**	3.321	1.083	to	10.181	**0.036**
Follow-up period	0.452	0.319	to	0.641	**< 0.001**	0.655	0.211	to	2.033	0.464
Average Phe per year	0.605	0.477	to	0.768	**< 0.001**	1.251	0.671	to	2.334	0.481
Control situation	0.351	0.186	to	0.660	**0.001**	1.342	0.498	to	3.609	0.560

Bold indicates statistical significance (*p* < 0.05)

*BMI* body mass index, *OR* odds ratio, 95% CI, 95% confidence interval

^*^P was calculated after adjusting for sex, region, income, paternal education level, and maternal education level

In Table 3, upon stratification by different levels of psycho-behavioral problems, factors identified as significant after multiple adjustment included BMI, age, pregnancy order, delivery order, disease diagnosis mode, and diet therapy pressure. Pregnancy order, delivery order, disease diagnosis mode, and diet therapy pressure were only significant in the severe group (compared to the without and slight groups) and not in the moderate group. In addition, Spearman correlation coefficients for the PSQ and HADS subscales indicated that age was correlated with conduct problems score, learning problems score, impulsive-hyperactivity score, and hyperactivity index score. In addition, pregnancy order was correlated with learning problems score, impulsive-hyperactivity score, and hyperactivity index score. Delivery order was correlated with conduct problems score, learning problems score, impulsive-hyperactivity score, hyperactivity index score, and depression score, as presented in Table 4.

**Table 3:** Identification of potential factors responsible for PKU children with different levels of psycho-behavioral problem

Subgroups	Model	Without or slight	Moderate	Severe
BMI	Model 1^a^	1 [Ref]	1.14 (0.99, 1.31)	1.08 (0.96, 1.23)
	Model 2^b^	1 [Ref]	1.10 (1.02, 1.28)^*^	1.13 (1.06, 1.32)^*^
Age	Model 1^a^	1 [Ref]	3.23 (1.02, 5.24)^*^	5.53 (1.39, 6.95)^*^
	Model 2^b^	1 [Ref]	1.95 (0.52, 7.38)	5.81 (1.34, 6.56)^*^
Pregnancy order	Model 1^a^	1 [Ref]	0.25 (0.03, 1.84)	0.33 (0.08, 1.38)
	Model 2^b^	1 [Ref]	0.12 (0.13, 1.18)	0.16 (0.03, 0.83)^*^
Delivery order	Model 1^a^	1 [Ref]	0.15 (0.06, 1.53)	0.16 (0.08, 1.26)
	Model 2^b^	1 [Ref]	0.14 (0.06, 1.14)	0.07 (0.01, 0.64)^*^
Highest Phe before treatment	Model 1^a^	1 [Ref]	1.01 (0.96, 1.05)	0.98 (0.94, 1.03)
	Model 2^b^	1 [Ref]	0.96 (0.95, 0.99)^*^	0.98 (0.93, 1.04)
Recent Phe	Model 1^a^	1 [Ref]	0.92 (0.78, 1.08)	1.02 (0.91, 1.13)
	Model 2^b^	1 [Ref]	0.93 (0.79, 1.08)	1.02 (0.91, 1.14)
Age of initial treatment	Model 1^a^	1 [Ref]	0.97 (0.93, 1.02)	0.98 (0.97, 0.99)^*^
	Model 2^b^	1 [Ref]	0.96 (0.92, 1.02)	0.99 (0.97, 1.01)
Delivery mode	Model 1^a^	1 [Ref]	6.69 (0.81, 9.57)	1.48 (0.46, 4.78)
	Model 2^b^	1 [Ref]	6.28 (0.74, 9.61)	1.44 (0.41, 5.02)
Disease diagnosis mode	Model 1^a^	1 [Ref]	0.74 (0.14, 3.77)	1.96 (0.62, 6.23)
	Model 2^b^	1 [Ref]	0.55 (0.10, 3.09)	2.00 (1.52, 7.69)^*^
Diet therapy pressure	Model 1^a^	1 [Ref]	2.49 (0.56, 5.13)	5.09 (1.53, 6.88)^**^
	Model 2^b^	1 [Ref]	2.40 (0.49, 5.71)	4.31 (1.09, 6.95)^**^
Follow-up period	Model 1^a^	1 [Ref]	0.60 (0.12, 3.06)	1.20 (0.37, 3.93)
	Model 2^b^	1 [Ref]	0.30 (0.05, 1.84)	0.96 (0.26, 3.67)
Average Phe per year	Model 1^a^	1 [Ref]	1.04 (0.45, 2.41)	1.28 (0.64, 2.58)
	Model 2^b^	1 [Ref]	1.05 (0.43, 2.58)	1.47 (0.67, 3.20)
Control situation	Model 1^a^	1 [Ref]	1.03 (0.27, 3.84)	1.17 (0.39, 3.56)
	Model 2^b^	1 [Ref]	1.12 (0.27, 4.64)	1.51 (0.45, 5.15)

Bold indicates statistical significance (*p* < 0.05)

*BMI* body mass index, *OR* odds ratio, 95% CI, 95% confidence interval

^a.^ Model 1: unadjusted model

^b.^ Model 2: adjusted for sex, region, income, paternal education level, and maternal education level

**Table 4:** Spearman correlations (r) and statistical significance (p) between six predictive variables^a^ and particular problems scores^b^

Predictive Variables	**BMI**		**Age**		**Pregnancy order**		**Delivery order**		**Disease diagnosis mode**		**Diet therapy pressure**	
Particular Problems Scores	r	p	r	p	r	p	r	p	r	p	r	p
Conduct problems score	0.000	0.999	0.305	**0.002**	-0.189	0.059	-0.226	**0.023**	-0.069	0.495	0.120	0.234
Learning problems score	0.031	0.756	0.260	**0.009**	-0.206	**0.039**	-0.225	**0.025**	-0.108	0.285	0.136	0.177
Psychosomatic issues score	0.170	0.092	-0.046	0.652	0.035	0.727	-0.037	0.719	-0.118	0.240	-0.152	0.128
Impulsive-hyperactivity score	-0.040	0.695	0.272	**0.006**	-0.250	**0.012**	-0.296	**0.003**	-0.084	0.407	0.139	0.168
Anxiety score	0.018	0.862	0.102	0.310	-0.037	0.714	-0.048	0.633	-0.006	0.954	0.059	0.562
Hyperactivity index score	0.067	0.505	0.281	**0.005**	-0.222	**0.027**	-0.269	**0.007**	-0.107	0.289	0.159	0.114
Anxiety score	0.113	0.264	0.129	0.199	-0.127	0.207	-0.178	0.077	0.063	0.534	0.154	0.126
Depression score	0.083	0.410	0.149	0.138	-0.146	0.146	-0.214	**0.033**	0.091	0.369	0.117	0.247

Bold indicates statistical significance (*p* < 0.05)

*BMI* body mass index

^a.^ Six Predictive Variables included BMI, Age, Pregnancy order, Delivery order, Disease diagnosis mode, and Diet therapy pressure

^b.^ Particular Problems included Conduct problems score, Learning problems score, Psychosomatic issues score, Impulsive-hyperactivity score, Anxiety score, Hyperactivity index score, Anxiety score, and Depression score

### Prediction accuracy assessment

**Fig. 2:**
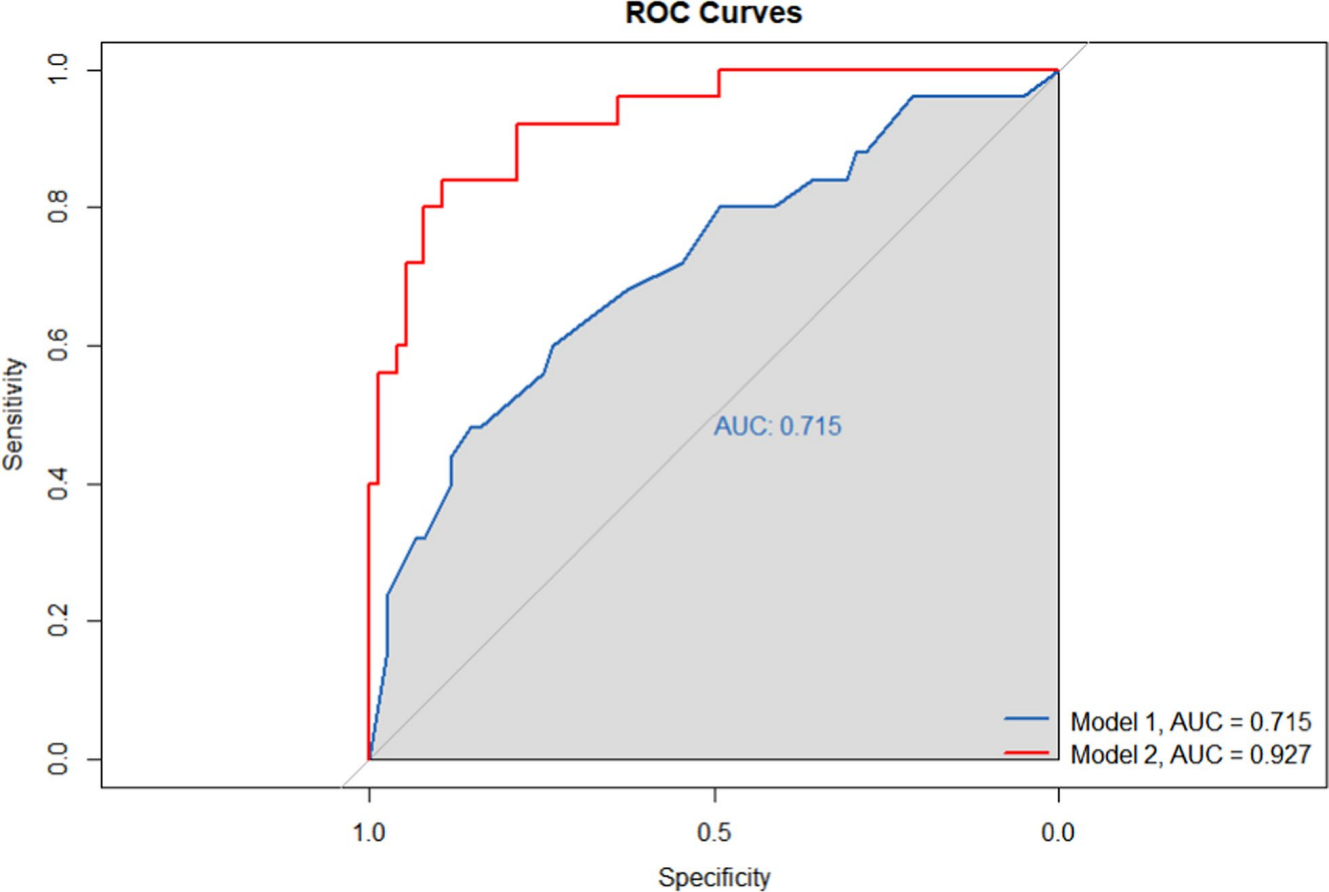
Receiver operating characteristic (ROC) curves for both basic model and full model. Abbreviations: AUC, area under curve. The blue solid line corresponds to the basic model, and the red solid line corresponds to the full model. Basic model included all variables under study with the exception of six significant factors identified in Table 2, and full model included all variables under study

Two models were constructed to evaluate the prediction performance of six significant factors: a basic model, which encompassed all, except for, the six significant factors, and a comprehensive full model, integrating all surveyed factors. To gauge model accuracy, a wide panel of calibration and discrimination metrics were calculated for both models. Notably, the full model exhibited marked enhancement in prediction accuracy over the basic model, as evidenced in Table 5**.** Moreover, the Area under the Receiver Operating Characteristic Curve (AUROC) analysis underscored statistically significant discrimination between the two models (*P* < 0.001), with the distinct outcomes vividly portrayed in the ROC curves (Fig. 2).

**Table 5:** Prediction accuracy gained by adding six significant factors identified for PKU children with psycho-behavioral problem

Statistics	Basic model	Full model
**Calibration**		
AIC	115.1776	81.9296
BIC	130.8086	113.1916
**Discrimination**		
AUROC	0.715	0.927
P value for AUROC	< 0.001	

*AIC* Akaike information criterion, *BIC* Bayesian information criterion, *AUROC* area under the receiver operating characteristic. Basic model included all variables under study with the exception of six significant factors identified in Table 2, and full model included all variables under study

### Risk prediction nomogram model

**Fig. 3:**
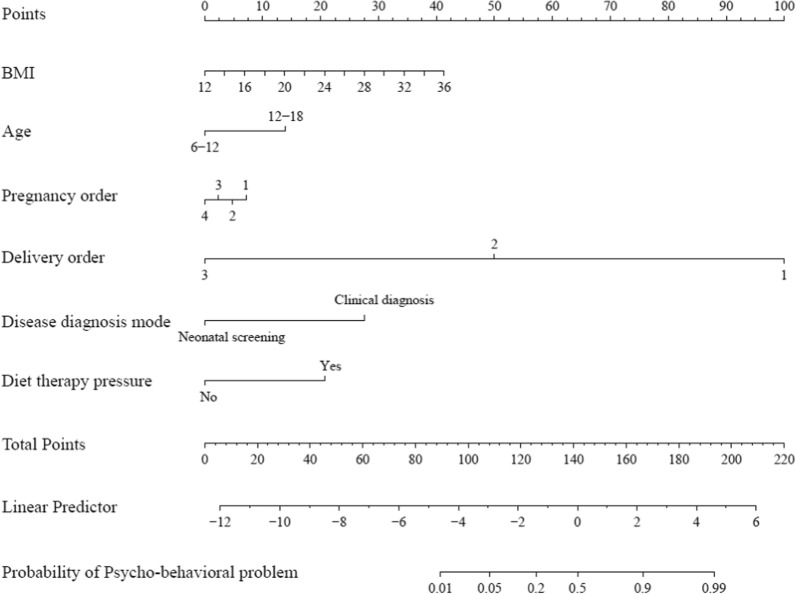
Prediction nomogram for the risk of having psycho-behavioral problems among PKU children. *Notes*. This nomogram is designed to predict the probability of psycho-behavioral problems based on several predictor variables. Each variable, including BMI (body mass index) (ranging from 12 to 36), age categories (6–12 and 12–18), pregnancy order (1 to 4), delivery order (1 to 3), disease diagnosis mode (neonatal screening or clinical diagnosis), and diet therapy pressure (yes or no), is assigned a specific number of points. The total points, which can range from 0 to approximately 220, are then used to determine the probability of a psycho-behavioral problem, ranging from 0.01 to 0.99. To use the nomogram, locate the value of each predictor on its respective scale to obtain the points, sum these points, and then use the total points to find the corresponding probability on the bottom scale

**Fig. 4:**
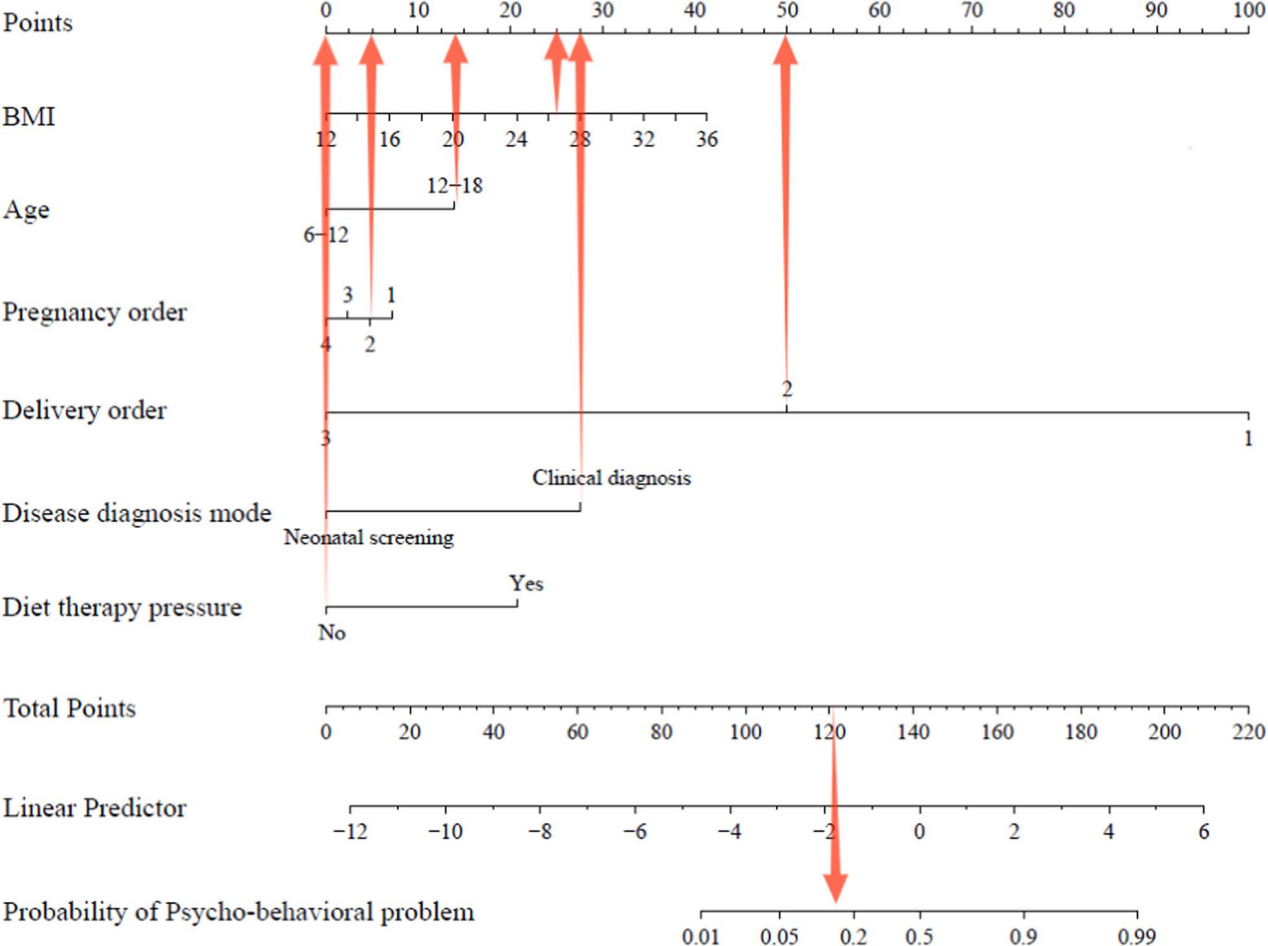
Example of prediction nomogram for psycho-behavioral problems among PKU children. For example, for a 13 years old child (14 points) with a BMI of 26.2 kg/m^2^ (25 points), pregnancy order of two (5 points), delivery order of two (50 points), clinical diagnostic confirmation of PKU (27.5 points), and without dietary therapy-related stress (0 points), the total score obtained from the nomogram was approximately 121.5 points. This composite score corresponds to an estimated 15% probability of developing psycho-behavioral problems

For practical reasons, a nomogram model was constructed, as shown in Fig. 3, to help intuitively predict the overall risk of psycho-behavioral problems in children with PKU by modeling promising and important influencing factors under study. The complete scoring methodology and parameter integration process are systematically presented in Fig. 4. For example, for a 13 years old patient (14 points) with a BMI of 26.2 kg/m^2^ (25 points), pregnancy order of two (5 points), delivery order of two (50 points), clinical diagnostic confirmation of PKU (27.5 points), and without dietary therapy-related stress (0 points), the total score obtained from the nomogram was approximately 121.5 points. This composite score corresponds to an estimated 15% probability of developing psycho-behavioral problems.

## Discussion

The aim of this study was to estimate the prevalence of psycho-behavioral problems among PKU children and explore potential factors associated with psycho-behavioral problems among 100 PKU children aged 6–18 years from China-Japan Friendship Hospital. The key finding of this study was the significant association between psycho-behavioral problems and factors including body mass index (BMI), age, birth order, pregnancy sequence, disease diagnosis method, and dietary therapy pressure. To our knowledge, this is the first study to date that evaluating contributing factors for psycho-behavioral problems among Chinese PKU children in their pre-adolescent and adolescent stages.

In this study, approximately 25% of Chinese children aged 6–18 years with PKU presented with psycho-behavioral problems. Beyond determining its prevalence rate, we endeavored to identify potential contributing factors underlying the emergence of psycho-behavioral problems among PKU-affected children, leveraging the insights garnered from our survey data. Overall, this study identified six factors, BMI, age, pregnancy order, delivery order, disease diagnosis mode, and diet therapy pressure that are significantly associated with psycho-behavioral problems in children with PKU. These factors may have a more pronounced influence on children with more severe symptoms.

First, we identified that BMI was a significant predictor of psychological and behavioral issues in Chinese children with PKU, findings that align with those of some longitudinal studies. For instance, Paterno et al. [23] demonstrated similar BMI between children with PKU and their non-affected peers, but noted greater variability within the PKU cohort. This variability might reflect suboptimal nutritional balance, which can indirectly exacerbate psychological stress stemming from dietary restrictions, body image concerns, and social stigma associated with the condition [24, 25]. Ahmadzadeh et al. [26] found in 80 children with PKU, the prevalence of obesity was 37.5% and that of being overweight was 43.8%, emphasizing the considerable weight-related burden in this patient population. A recent meta-analysis [27] showed that subgroups with classic PKU exhibited significantly higher BMI, challenging the assumption that phenylalanine-restricted diets inherently promote weight gain. The exact mechanisms underlying the comorbidity between PKU and obesity remain underexplored, yet both conditions share common psychological risk profiles, including depression, anxiety, and social dysfunction. Existing research on PKU has predominantly focused on direct neurological impacts, whereas studies on obesity have placed emphasis on psychosocial aspects. To further our understanding in this field, future research should prioritize the metabolic-psychological interactions between PKU and weight-related outcomes.

Second, age emerged as a critical factor in our study, and it exerted a profound influence on psycho-behavioral outcomes among adolescents with PKU, in line with prior research emphasizing age-related challenges in PKU management [28–30]. For instance, studies by Manti et al. [28, 29] and Esgi et al. [28, 29] reported declining adherence to dietary regimens during adolescence, disease burden, and elevated blood phenylalanine level, which in turn affected brain development and increased the incidence of mental diseases. A longitudinal retrospective study by Garcia et al. [30] showed that most patients transition from childhood to young adulthood and even adulthood, and the management of this transition period is particularly difficult. Extending the results of prior studies providing qualitative estimates, we quantified the magnitude of age-related risk and established a connection between these risks and metabolic imbalances.

Third, diet therapy pressure identified in our study underscores the multifaceted psychosocial challenges faced by PKU patients and their families. These stressors encompass the continuous necessity of meticulously calculating phenylalanine intake to avoid metabolic dysregulation, restrictions on social activities (e.g., difficulty participating in shared meals or celebrations due to reliance on special medical foods), psychological distress linked to procuring such foods (e.g., financial burden and availability issues), or persistent worries about long-term micronutrient deficiencies despite strict dietary adherence. This is in accordance with findings of Alaei et al. [31], who reported that parental unemployment was significantly associated with poorer metabolic control (elevated blood phenylalanine levels), likely due to constrained access to subsidized or free specialized dietary products. Financial barriers can exacerbate non-adherence, particularly in families with inadequate socioeconomic support, ultimately amplifying stress for both caregivers and patients. Moreover, the psychosocial strains associated with dietary restrictions may exacernate preexisting difficulties, such as social isolation or stigma related to atypical eating habits [30, 32]. Consequently, integrating psychological support systems tailored for PKU management, including counseling for coping strategies, financial assistance programs, and peer support networks, is critical to boosting treatment adherence and the quality of life of affected children and their families [3, 5, 33].

Fourth, our finding that clinical diagnosis (vs. neonatal screening) increases psycho-behavioral risk is in line with evidence emphasizing the critical role of early diagnosis. Neonatal screening programs are instrumental in averting irreversible neurological damage by facilitating swift dietary intervention. Despite their implementation globally, a portion of children remain undiagnosed during the neonatal period and are identified clinically at older ages due to emerging symptoms. This diagnostic gap can be attributable to a multitude of factors. Limitations in screening protocols, such as false negatives from premature blood sampling before phenylalanine accumulation, regional disparities in healthcare resources, or familial refusal to participate in screening programs played a part [34, 35]. Studies confirmed that delayed diagnosis correlated with extended exposure to elevated phenylalanine levels, which in turn worsens neurodevelopmental deficits and psychiatric comorbidities [6, 33]. For instance, children diagnosed post-symptomatically often exhibit more severe cognitive impairments and emotional dysregulation, presumably due to irreversible neuronal damage caused from chronic hyperphenylalaninemia. Our data further corroborate the critical role of early screening in mitigating long-term psychosocial sequelae and underscore the necessity of addressing systemic and sociocultural barriers to achieve universal neonatal screening coverage.

Fifth, our findings indicated that both the sequence of pregnancy and delivery were recognized as important factors. Although no prior studies on PKU have explicitly address this aspect, evidence from genetic counseling suggests that families with affected children often pursue rigorous prenatal testing in subsequent pregnancies [1, 7, 36]. This heightened level of vigilance may lead to enhanced parental awareness and personalized care, potentially alleviating the psychosocial stressors associated with raising a child with PKU. However, it is important to note that this hypothesis remains speculative, and warrants further investigations through longitudinal studies or controlled trials to elucidate the causal mechanisms underlying these observed associations.

Finally, to enhance the applied value of our findings, we employed nomogram techniques to develop a predictive model for psycho-behavioral problems in PKU children. The nomogram we constructed integrates multiple predictor variables, including BMI, age, pregnancy order, delivery order, disease diagnosis mode, and diet therapy pressure, to provide a comprehensive and user-friendly risk assessment tool. The overall accuracy of the nomogram prediction model was good, suggesting its potential utility in clinical practice. Healthcare professionals can use the nomogram to easily estimate an individual patient’s risk of psycho-behavioral problems based on specific characteristics. This enables a more personalized and targeted approach to patient management, potentially improving outcomes. Despite the apparent associations and the excellent accuracy of the nomogram, our findings are preliminary. Validation in other independent, well-designed longitudinal studies is needed to confirm or refute our conclusions. Future research should focus on validating the nomogram in diverse populations and settings, as well as exploring its application in other related metabolic disorders.

## Limitations

This study has several strengths. First, due to the rarity of PKU, the inclusion of a large sample size and adoption of multifaceted exploration approach are major advantages. This in-depth analysis offers valuable insights and serves as a reliable reference for clinicians in diagnosing and managing childhood PKU. Second, using tools like the PSQ and HADS enhances the study’s comprehensiveness. These instruments efficiently screen for various mental, psychological, and behavioral concerns in children, enabling prompt identification and targeted interventions.

Some limitations should be acknowledged for this study. First, relying on parental-reported data can introduce bias, particularly for younger children or those with cognitive impairments, as parents’ perceptions may not fully align with their children’s actual experiences. Although we tried to address discrepancies by involving a second guardian when feasible, subjective interpretations could still undermine the accuracy of our results. Second, the cross-sectional design of this study constrains our ability to establish definitive causal relationships between potential factors and psycho-behavioral problems in PKU. Third, this study is limited by the lack of detailed data on some key variables, such as number of siblings within families, whether any siblings have PKU, and the residual phenylalanine hydroxylase activity levels of participants. Future research should strive to collect more comprehensive datasets with these variables, and enable a more nuanced understanding of the complex psycho-behavioral outcomes in this population. Fourth, although our approach prioritized feasibility within a rare-disease cohort, we recognize that having psychologist directly involved in data collection could deepen behavioral phenotype analysis. Future studies could integrate psychological co-investigators to explore mechanistic links between metabolic control and neurocognitive outcomes.

## Conclusions

Our findings indicate that approximately one in four PKU children aged 6–18 exhibited psycho-behavioral problems. Additionally, we have uncovered six factors that show a notable correlation with these problems in PKU. These insights shed light on key risk factors for psycho-behavioral challenges in PKU-affected children, potentially informing the development of targeted interventions to mitigate their occurrence.

## Data Availability

Data are available upon reasonable requirements.
